# Flexible Transient Resistive Memory Based on Biodegradable Composites

**DOI:** 10.3390/nano12193531

**Published:** 2022-10-09

**Authors:** Lu Wang, Yukai Zhang, Peng Zhang, Dianzhong Wen

**Affiliations:** Heilongjiang Provincial Key Laboratory of Micronano Sensitive Devices and Systems, School of Electronic Engineering, Heilongjiang University, Harbin 150080, China

**Keywords:** physical transient, biological materials, graphene quantum dots, “OR gate”

## Abstract

Physical transient electronics have attracted more attention as the basis for building green electronics and biomedical devices. However, there are difficulties in selecting materials for the fabricated devices to take into account both biodegradability and high performance. In this paper, a physically transient resistive random-access memory (RRAM) device was fabricated by using egg protein and graphene quantum dot composites as active layers. The sandwich structure composed of Al/EA:GQD/ITO shows a good write-once-multiple-read memory characteristic, and the introduced GQD improves the switching current ratio of the device. By using the sensitivity of GQDs to ultraviolet light, the logic operation of the “OR gate” is completed. Furthermore, the device exhibits a physical transient behavior and good biodegradability due to the dissolution behavior in deionized water. These results suggest that the device is a favorable candidate for the construction of memory elements for transient electronic systems.

## 1. Introduction

With significant strides in technology, a large number of electronic products have been produced [1], but these electronic products usually have the problem of being difficult to degrade [2]. Whether they are incinerated or buried, they will have different degrees of impact on the environment. The growing concern for environmental protection has promoted the research and development of advanced transient electronic devices [3,4,5]. These transient electronics can degrade without affecting the environment to reduce environmental pollution. As an important part of electronic devices, memory devices, when combined with transient characteristics, promise to have the abovementioned advantages [6,7]. Resistive random-access memory has the advantages of a high density, faster running speed, and lower power consumption and has become a favorable candidate for the next generation of nonvolatile memory [8,9,10,11,12,13]. RRAM prepared from natural biological materials mainly includes gelatin [14,15], starch [16], DNA [17,18], ferritin [19,20], chitosan [21,22], and so on. As a natural biological material, protein has good properties and application potential. It has been reported that the use of proteins to prepare transistors, field-effect transistors, nonvolatile RRAM, and so on [23,24,25,26]. Egg protein is a natural material that occurs widely in nature. Its composition is 10% protein and 90% water, and the proteins are mainly ovalbumin, mucin, and globulin. Egg protein is also biodegradable, biocompatible, and environmentally friendly, so it is useful to explore its application in electronic products. According to reports, egg protein has been used as a bound layer to fabricate RRAM and thin-film transistors [27,28]. The stepwise adjustment of the switching current ratio was accomplished by doping carbon nanotubes; the UV fluorescence was enhanced by doping gold nanoparticles [29]. Soluble memristors were fabricated with soluble electrodes Mg and W as upper and lower electrodes, respectively, and egg protein as a dielectric layer [30]. Although considerable research has been achieved, more work is still needed to further explore the internal conduction mechanism of the device and improve the performance and reliability of the device [31].

Although graphene is an attractive new 2D material, its zero band gap makes it unsuitable for flexible storage applications [32]. Graphene quantum dots (GQDs) have attracted extensive attention due to their many excellent properties, such as edge states and variable quantum confinement effects, as well as broad application prospects. In applications, GQDs have the potential to replace commonly used inorganic hybrid materials, including toxic and expensive heavy metals [33,34]. Compared with other nanoparticles, graphene quantum dots are considered suitable for flexible electronic storage devices due to their remarkable chemical inertness, higher electrical conductivity, and higher stability [35,36]. Furthermore, the edge states and quantum confinement effects of GQDs yield some physical and chemical properties suitable for charge trapping [37]. A typical bipolar resistive switching behavior was demonstrated by fabricating an RRAM with the structure of Ag/GQD/TiO_x_/FTO [38]. For the RRAM fabricated by the composite of multiwalled carbon nanotubes and GQDs, the switching current ratio of the device can be adjusted by adjusting the concentration of GQDs and applying UV light [39].

Currently, the focus of bioelectronics is mainly on compatibility and inherent performance. Traditional electronic products are usually composed of semiconductor materials and electrodes, which are generally insoluble or a relatively small part of it. Therefore, their application as biodegradable devices is limited. In this work, using the natural biomaterial egg protein as the active layer, the device exhibits a write-once-read-many memory property. By doping graphene quantum dots, the switching current ratio of the device is improved. Using the sensitive properties of graphene quantum dots to ultraviolet light, the logic operation of the “OR gate” in the logic gate is completed. Among them, the active layer can be completely dissolved in deionized water within 20 min, and the device cannot completely switch the resistance state. The results show that the device has good physical transient performance.

## 2. Materials and Methods

### 2.1. Preparation of the Device

First, the PET substrate (the thickness of substrate is 0.175 mm) coated with the ITO electrode was placed in acetone, absolute ethanol, and deionized water for ultrasonic cleaning, and the cleaning time was 10 min each time to remove the oil stains attached to the surface of the ITO electrode. Eggs were purchased from local supermarkets. We used a dropper to separate the egg whites and yolks. Untreated proteins have high viscosity and poor film-forming properties during device fabrication. Therefore, the protein and deionized water were diluted at a volume ratio of 1:10 and sonicated for 15 min to thoroughly mix the protein and deionized water. The diluted protein solution and graphene quantum dots (purchased from the manufacturer Suzhou Hengqiu Technology, Suzhou, China, concentration 1 mg/mL, purity 80%, average diameter 15 nm, thickness 0.5–2.0 nm) were mixed at a volume ratio of 3:1. Similarly, the mixed solution was subjected to ultrasonic treatment for 15 min so that the two were mixed evenly to obtain an EA:GQD composite material. The EA:GQD composites were spin-coated on the ITO electrode-coated PET substrate and rotated at a low speed of 500 rpm for 5 s and at a high speed of 4000 rpm for 20 s. Then, the active layer was dried at 80 °C for 10 min. Finally, the aluminum top electrode was evaporated on the active layer by the thermal evaporation method to complete the preparation of the whole device.

### 2.2. Characteristic Test

Under the conditions of normal temperature and pressure, the electrical properties of the RRAM of the Al/EA:GQD/ITO/PET device were tested with a semiconductor parametric tester (Keithley 4200) (Keithley, Solon, OH, USA). The cross sections of the devices were characterized by scanning electron microscopy (Hitachi S-3400 N, Hitachi, Tokyo, Japan). The UV light power was 171.42 mW/cm^2^ and the wavelength was 395 nm.

## 3. Results

**Figure 1:**
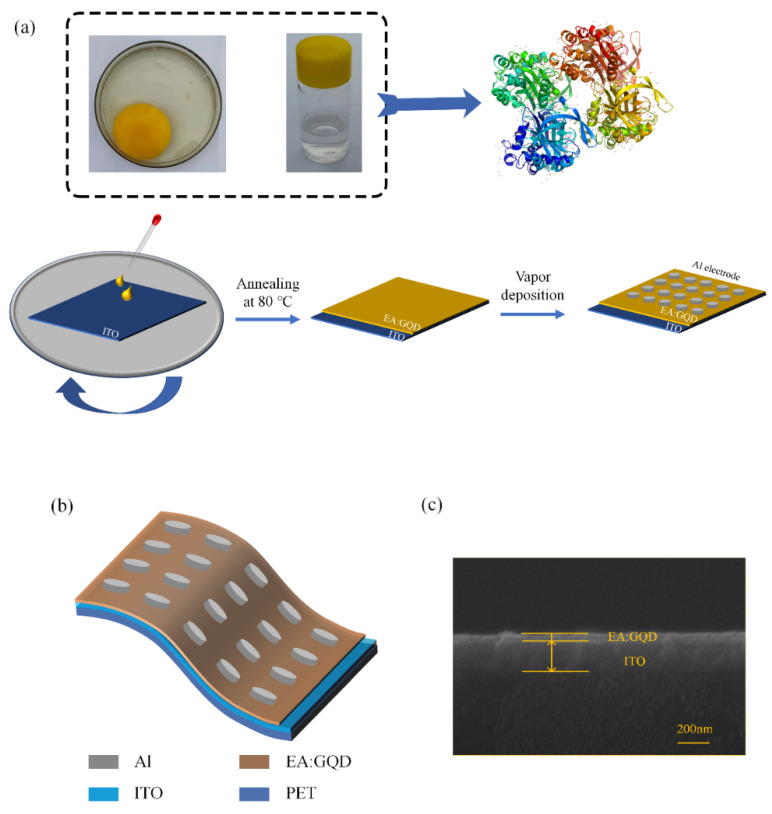
Al/EA:GQD/ITO/PET device: (**a**) fabrication flow chart. (**b**) Structure schematic and (**c**) SEM of the active layer.

As the main component of the active layer of the device, egg protein isolated from eggs was dissolved in deionized water, after which graphene quantum dots were doped, and the solution was spin-coated on ITO electrodes by spin coating and thermal evaporation. The preparation of the device was completed by the method, and the specific preparation process is shown in Figure 1a. The schematic structure of the device Al/EA:GQD/ITO/PET is shown in Figure 1b, from top to bottom corresponding to the top Al electrode, the active layer EA:GQD, and the bottom electrode ITO. The cross-sectional thickness and morphology of the active layer and bottom electrode ITO of the device were characterized by scanning electron microscopy. The results are shown in Figure 1c. The thickness of the ITO electrode of the device is approximately 200 nm, and the thickness of the active layer EA:GQD is approximately 50 nm. In the SEM images, it can be seen that the active layer of the device has a high flatness.

**Figure 2:**
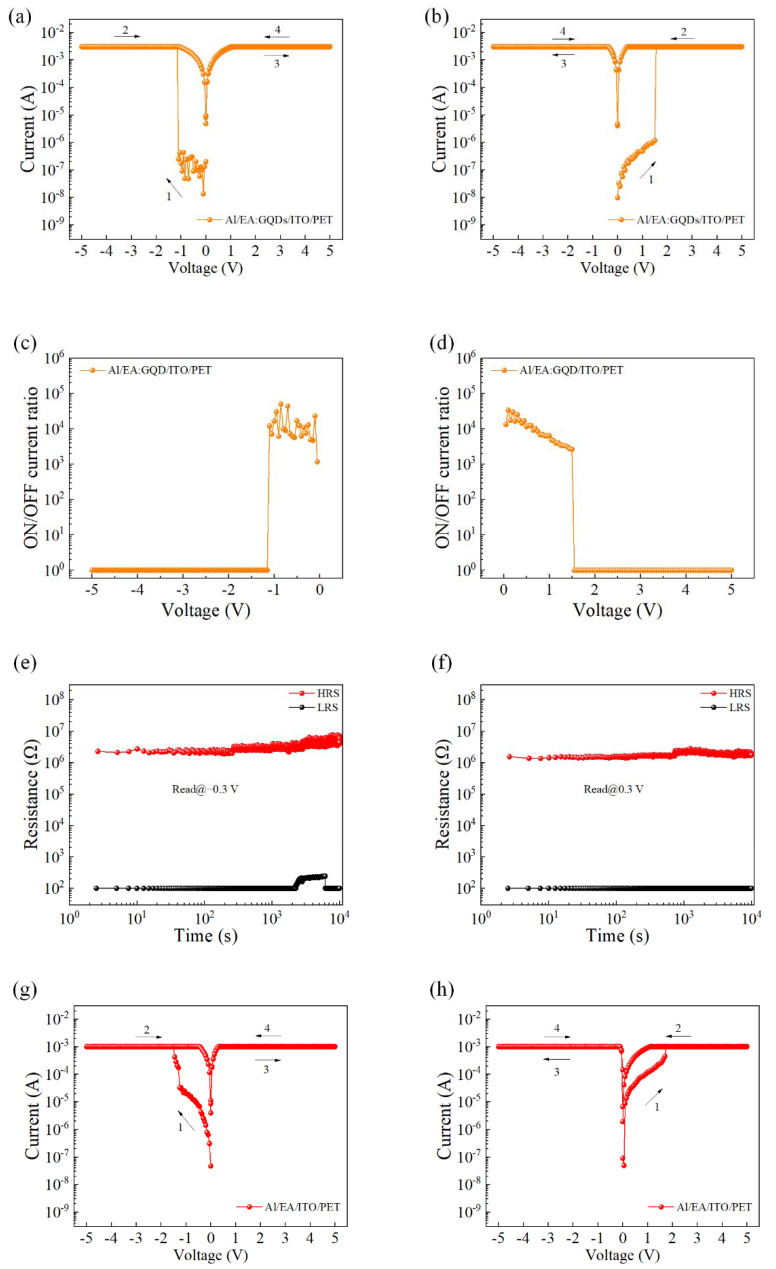
The *I–V* characteristic curve of the Al/EA:GQD/ITO/PET device: (**a**) initial negative voltage and (**b**) initial positive voltage. Switching current ratio (**c**) initially applied negative voltage and (**d**) initially applied positive voltage. Hold time, read voltage (**e**) −0.3 V (**f**) 0.3 V. *I–V* characteristic curve of device Al/EA/ITO/PET. (**g**) Initial negative voltage applied. (**h**) Initial positive voltage applied.

The electrical properties of the EA:GQD-based flexible devices were tested at room temperature and pressure. During the test, the bottom electrode ITO was always kept grounded, and an external scanning voltage was applied to the top Al electrode. The device exhibited typical write-once-read-many characteristics, resistance state switching could occur at a specific voltage, and no resistance state switching occurred in the subsequent scanning process. Figure 2a,b shows the *I–V* characteristic curve of the Al/EA:GQD/ITO/PET device. From the figure, it can be found that the preparation of the device did not require the process of electroforming, and resistance switching could be performed directly. The magnitude of the device limiting current was set to 3 mA to prevent permanent breakdown of the device. An external bias voltage was applied on the upper electrode, while the lower electrode ITO was grounded. The switching current ratio of the device is shown in Figure 1c,d, and the average switching current ratio of the device was calculated to be 1.19 × 10^4^ through the results. The results showed that doping GQDs in the active layer could improve the switching current ratio of the device, which was increased by two orders of magnitude compared with the device with EA as the active layer. In practical circuit applications, a higher switch current ratio can improve the accuracy of identifying high- and low-resistance states. The test time was set to 10^4^ s, and the sampling interval was set to 2.50 s. As shown in Figure 2e,f, the resistance values of the device in the high- and low-resistance states were tested under read voltages of −0.30 V and 0.30 V, respectively. The state of the device remained stable and lasted for more than 10^4^ s. Therefore, the retention capability of the flexible Al/EA:GQD/ITO/PET device was proven to be reliable. Figure 2g,h shows the *I–V* characteristic curves of the device Al/EA/ITO/PET initially applied with a negative voltage and a positive voltage, respectively. Similarly, the initial resistance state of the device was HRS. After the external voltage reached the write voltage, the resistance state of the device was switched from HRS to LRS, and LRS was maintained in the subsequent three scans.

**Figure 3:**
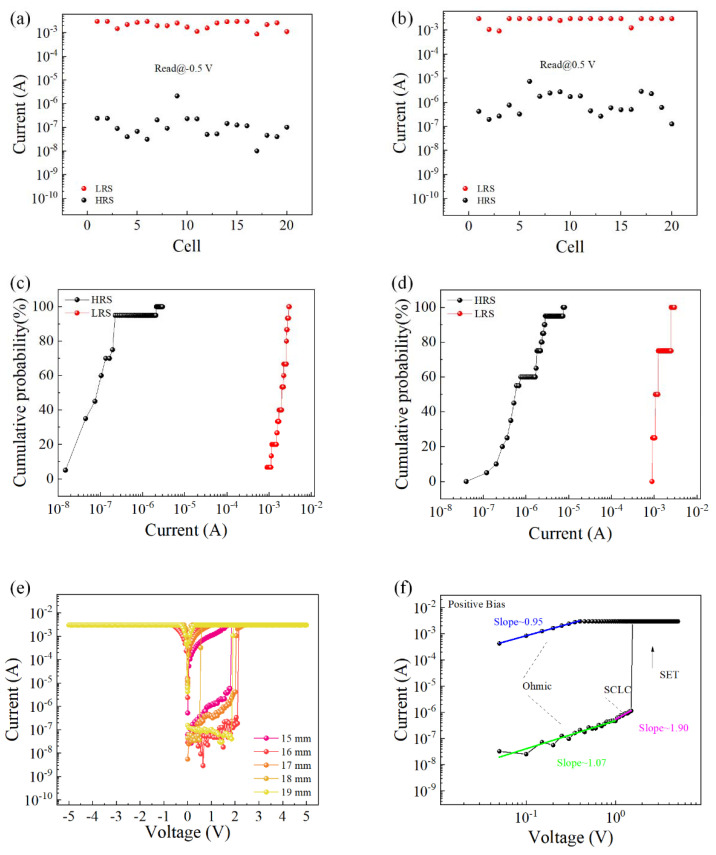
High and low resistance values of Al/EA:GQD/ITO devices. (**a**) Initially applied negative voltage. (**b**) Initially applied positive voltage. (**c**) Cumulative probability of initial negative voltage application. (**d**) Cumulative probability of initial positive voltage application. (**e**) *I–V* characteristic curves of different bending degrees. (**f**) Double logarithmic curve.

To verify the overall yield of the device, the *I–V* characteristic curves of all 20 cells on the device were tested, and the resistance values of the corresponding high- and low-resistance states were read under a fixed voltage value. As shown in Figure 3a, when a negative voltage was initially applied, the coefficient of variation in the high-resistance state was 2.13, and the coefficient of variation in the low-resistance state was 0.32; as shown in Figure 3b, when a positive voltage was initially applied, the high-resistance coefficient of variation in the state was 1.21, and the coefficient of variation in the low-resistance state was 0.26. Figure 3c,d is the corresponding cumulative probability of resistance. The *I–V* characteristic curves of the device under different bending degrees were tested, as shown in Figure 3e. When the flexible device Al/EA:GQD/ITO/PET (device size was 20 mm × 20 mm) was gradually bent from the flat state until the diameter of the device reached 15 mm, the device could still maintain normal operation, showing the WORM characteristic. When the degree of bending continued to increase, the device was damaged. This showed that the device could work normally in the bending state and maintain excellent bending stability over a large bending range. To understand the current transport mechanism in the device, Figure 3f shows the results of *I–V* characteristic curve fitting when a positive voltage was initially applied. The *I–V* curves of the writing process were redrawn in log–log coordinates. At low voltages, the fitted slope of the *I–V* curve was approximately 1, indicating that the current transport mechanism was governed by Ohm’s law. When the voltage increased, the relationship I = αV + βV^2^ was satisfied. At low voltage, the number of injected carriers was smaller than that of thermally generated carriers. Therefore, the conduction mechanism of the device was consistent with the trapping-controlled SCLC.

**Figure 4:**
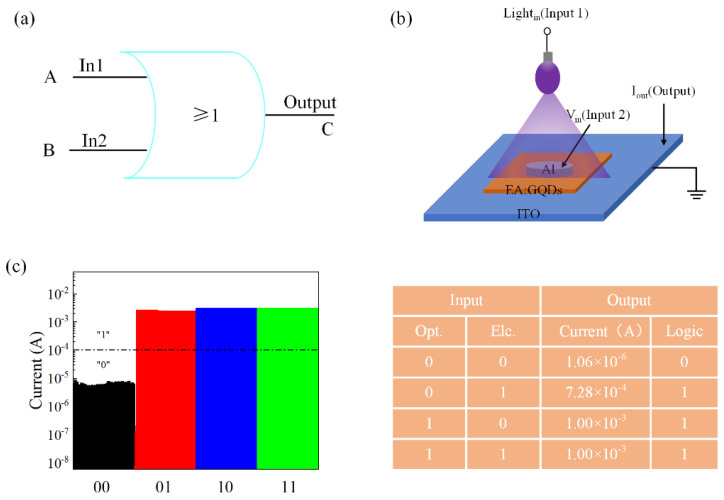
(**a**) OR gate. (**b**) Schematic diagram of applied illumination. (**c**) Current response of the device unit under different input signals.

Figure 4a shows an OR gate with a logical AND function. The two terminals of “A” and “B” are input terminals for the electrical signal and optical signal, respectively. Figure 4b is a schematic diagram of the device when applying UV light. When signals are input to the logic devices, they are output from the “C” terminal after processing. It can be concluded that a single abovementioned device can realize the OR gate function, thereby reducing the complexity of integration and circuit consumption. Based on the sensitivity of graphene quantum dots to UV light, Figure 4c shows the current response values of the Al/EA:GQD/Al/PET device in the optical signal, electrical signal, and optical and electrical signal. A device with an output current greater than or equal to 10^−4^ A corresponds to a logic value of “1”, and less than 10^−4^ A, the logic state corresponds to a “0”.

The test results showed that when a single electrical signal or a single optical signal was input to the device, the current of the device was approximately 1.00 × 10^−3^ A, corresponding to the logic state “1”. In addition, the photoelectric time signal was applied to the device together, and the output current was approximately 1.08 × 10^−3^ A, which also corresponded to state “1”. From the table, it can be concluded that a single device can complete the operation of a logical “OR gate”.

**Figure 5:**
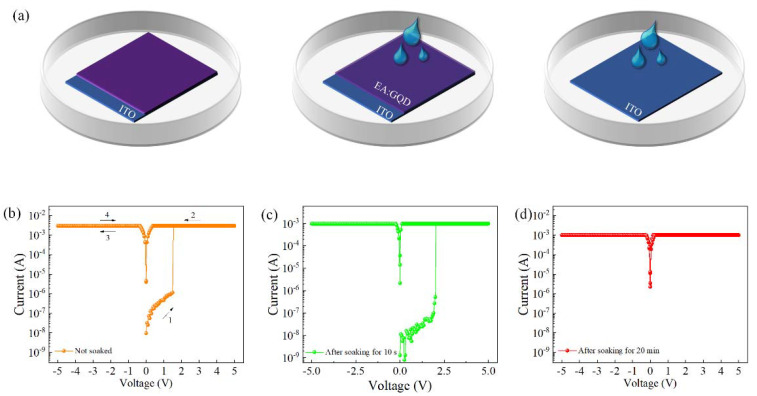
(**a**) Schematic diagram of the device placed in deionized water; *I–V* characteristic curves of the Al/EA:GQD/ITO/PET device. (**b**) Without soaking. (**c**) After soaking for 10 s. (**d**) After soaking for 20 min.

The prepared egg protein–graphene quantum dot active layer was placed in deionized water to test the physical transient properties of the device. A schematic diagram of the device placed in deionized water is given in Figure 5a. The device was placed in a Petri dish filled with deionized water to observe the changes in its active layer thin film. It can be seen from the schematic diagram that the initial active layer film had little change, and after 20 min, the edge layer was completely dissolved.

Due to the drying and curing process, the transparent active layer film did not undergo significant changes in the first few seconds of immersion. After 20 min, it could be clearly seen that the active layer disappeared through illumination. As shown in Figure 5b–d, the *I–V* characteristic curves of the device after soaking for 10 s and 20 min without soaking showed that the active layer was completely dissolved after soaking for 20 min, accompanied by the disappearance of electrical characteristics, showing the physical transient properties of the device.

**Figure 6:**
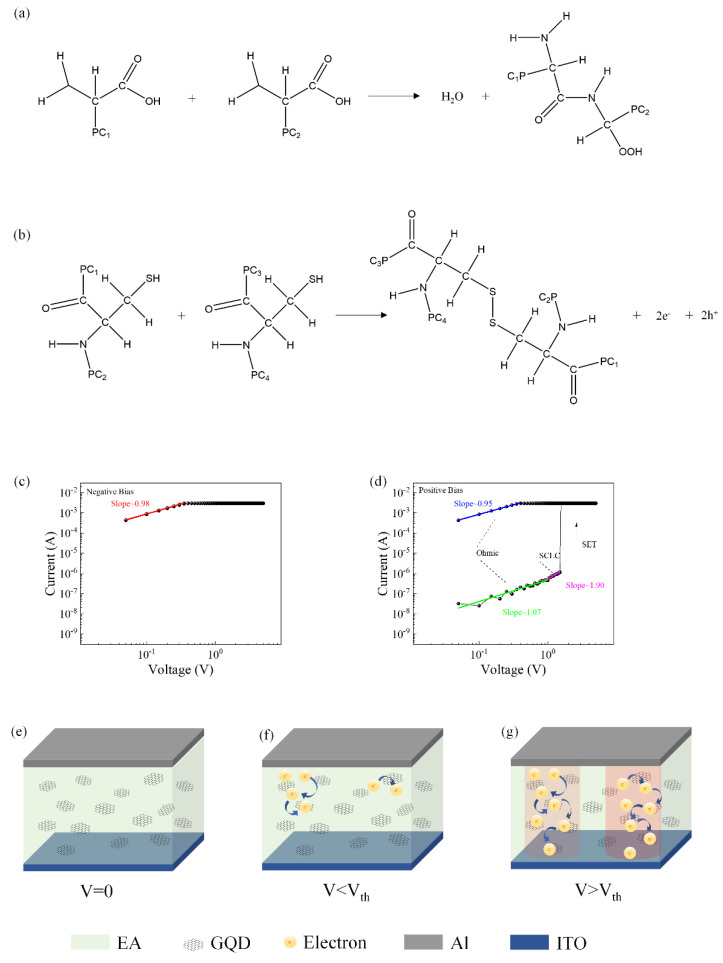
(**a**) Peptide bond formation process. (**b**) Disulfide bond formation process *I–V* curves of the device Al/EA:GQD/ITO/PET redrawn in double logarithmic coordinates. (**c**) Initially applied negative voltage. (**d**) Diagram of the conduction mechanism of the device with an initial positive voltage applied. (**e**–**g**) Diagram of the conduction mechanism.

Most of the proteins in egg whites are globular proteins composed of amino acids with long protein chains. Native proteins are held together by many weak chemical bonds. During the heating process, weak bonds are broken, and protein molecules are cross-linked with two main chemical bonds, peptide bonds, and disulfide bonds. The formation of disulfide bonds is an irreversible process and is responsible for the formation of solid protein membranes. Figure 6a,b shows the main processes of these two bond formations.

To understand the current transport mechanism in the device, the typical *I–V* characteristic curve of the device was redrawn on a logarithmic scale. Figure 6c,d shows the *I–V* characteristic curves in logarithmic coordinates and the fitting results when a positive voltage was initially applied. When the device was in the LRS, the slope of the fitted curve was approximately 1, which was the ohmic conduction behavior. When the device was in HRS, in the low voltage region, the fitted slope of the *I–V* curve was approximately 1, indicating that the current transport mechanism was governed by Ohm’s law. When increasing the voltage, the slope satisfied the relation I = αV + βV^2^. Therefore, the conduction mechanism of the device was consistent with trap-controlled SCLC.

The Al/EA:GQD/ITO/PET device exhibited the memory behavior of WORM. The proposed conduction mechanism based on the space-charge-confined current (SCLC) theory is shown in Figure 6e–g. In this device, egg protein served as the active layer, while graphene quantum dots served as charge-trapping sites embedded in the active layer. When a voltage was applied, electrons were injected into the organic blend film, and the traps were filled with the injected carriers. When the voltage reached the threshold voltage (V_set_), the carriers filled the traps completely, resulting in an exponential increase in the carriers in the active layer and the formation of conductive paths. The device switched from the HRS to the LRS. Due to the accumulation of a large number of electrons in the active layer, an internal electric field was formed. When a reverse voltage was applied to the device, due to the existence of the internal electric field, the charges in the traps were difficult to release, and the conduction path was not broken. Therefore, the device exhibited a write-once-read-many memory effect.

## 4. Conclusions

In this paper, a physical transient organic RRAM based on an Al/EA:GQD/ITO sandwich structure was demonstrated. The device exhibited good biodegradability and stable write-once-read-many memory performance, making it an ideal device for transient electronic systems. Combining the properties of the active layer and the properties provided by the double logarithmic curve, a conduction mechanism based on graphene quantum dots was proposed. For flexibility testing, the device still maintained good electrical properties at different degrees of bending. The active layer could be completely dissolved in deionized water within 20 min, indicating that the device had a controllable transient behavior and great potential for applications in bioelectronics.

## Data Availability

The data presented in this study are available on request from the corresponding author.
